# A Sustainable Approach to Asthma Diagnosis: Classification with Data Augmentation, Feature Selection, and Boosting Algorithm

**DOI:** 10.3390/diagnostics14070723

**Published:** 2024-03-29

**Authors:** Zne-Jung Lee, Ming-Ren Yang, Bor-Jiunn Hwang

**Affiliations:** 1 Department of Electronic and Information Engineering, School of Advanced Manufacturing, Fuzhou University, Quanzhou 362200, China; 2 Graduate Institute of Biomedical Informatics, College of Medical Science and Technology, Taipei Medical University, Taipei 235, Taiwan; tmu_ymz30@tmu.edu.tw; 3 College of Information Science, Ming Chuan University, Taoyuan 333, Taiwan; bjhwang@mail.mcu.edu.tw

**Keywords:** asthma, data augmentation, feature selection, extreme gradient boosting algorithm, generative adversarial networks

## Abstract

Asthma is a diverse disease that affects over 300 million individuals globally. The prevalence of asthma has increased by 50% every decade since the 1960s, making it a serious global health issue. In addition to its associated high mortality, asthma generates large economic losses due to the degradation of patients’ quality of life and the impairment of their physical fitness. Asthma research has evolved in recent years to fully analyze why certain diseases develop based on a variety of data and observations of patients’ performance. The advent of new techniques offers good opportunities and application prospects for the development of asthma diagnosis methods. Over the last few decades, techniques like data mining and machine learning have been utilized to diagnose asthma. Nevertheless, these traditional methods are unable to address all of the difficulties associated with improving a small dataset to increase its quantity, quality, and feature space complexity at the same time. In this study, we propose a sustainable approach to asthma diagnosis using advanced machine learning techniques. To be more specific, we use feature selection to find the most important features, data augmentation to improve the dataset’s resilience, and the extreme gradient boosting algorithm for classification. Data augmentation in the proposed method involves generating synthetic samples to increase the size of the training dataset, which is then utilized to enhance the training data initially. This could lessen the phenomenon of imbalanced data related to asthma. Then, to improve diagnosis accuracy and prioritize significant features, the extreme gradient boosting technique is used. The outcomes indicate that the proposed approach performs better in terms of diagnostic accuracy than current techniques. Furthermore, five essential features are extracted to help physicians diagnose asthma.

## 1. Introduction

Asthma is a chronic inflammatory condition of the respiratory tract. Clinically, it is characterized by frequent coughing, wheezing, shortness of breath, chest tightness, sputum, and asthma-related disorders such as rhinitis, sinusitis, nasal polyps, and atopic dermatitis. In severe cases, it will cause chest tightness, breathing difficulties, and even the feeling of not being able to breathe in air. Asthma is a global health problem. In addition to high mortality, asthma also causes significant economic losses due to the deterioration of the quality of life and the decline in the physical fitness of patients [1]. Asthma prevalence has risen substantially over the last few decades, with more than 300 million people affected, which is estimated to reach 400 million by 2025 [2]. Asthma symptoms vary and can be classified as annual attacks, seasonal attacks, or a combination of the two, and can also be classified according to persistent, paroxysmal, intermittent, or persistent evidence plus acute attacks, the length of the attack, the frequency, the changes between night and day, and the symptoms at night. The pathogenesis of asthma, including genetic, environmental, infectious, and nutritional factors, is still poorly understood [3]. Therefore, the World Health Organization (WHO) encourages research teams to investigate asthma symptoms and pathogenesis to develop new strategies for the defense and treatment of asthma and to stimulate the development of new treatments.

Traditionally, technologies like data mining and machine learning have been used to diagnose diseases. These techniques include particle swarm optimization (PSO), support vector machines (SVMs), random forests, automatic associative memory neural networks, backpropagation networks, Bayesian networks, and decision trees. Elkhenini et al. suggested a unique data mining method based on semantic analysis to extract data on chronic obstructive pulmonary disease (COPD) and asthma from two different databases in England [4]. Thus, specific information could be obtained from a massive amount of case data using information retrieval techniques with appropriate standards. Tsang et al. exploited the mHealth dataset published by the Asthma Mobile Health Research (AMHS) Center and applied machine learning techniques to construct early warning algorithms for asthma diagnosis [5]. Prasad et al. diagnosed asthma using questionnaires and medical data with a two-phase model. The first phase collects data from healthy people, while the second part collects data from asthmatic patients [6]. Ansari et al. introduced the Adaptive Fuzzy Inference System (ANFIS), which reduces asthma diagnostic errors through a backpropagation technique [7]. However, there is frequently an imbalance in asthma data, which means that the characteristics of healthy people (majority class) may predominate over asthma symptoms (minority class) [8]. Traditional algorithms frequently divide the minority class into the majority class when there is an imbalance in the asthma data, achieving a high accuracy rate; however, this makes it difficult for the algorithm to classify important minority samples [9].

In the data on asthma symptoms, there is frequently an imbalance problem. Recently, generative adversarial networks (GANs) have been shown to provide new data augmentation solutions for the imbalance problem [10,11]. Moreover, there are numerous features for data on asthma. Through the link between features, feature selection can reduce dimensionality and enhance learning models. However, GAN training is unstable and it is difficult to quantitatively determine when the generated network will generate high-quality data. Furthermore, the integration of data augmentation and feature selection is also an important issue. Extreme gradient boosting (XGBoost) uses machine learning algorithms within the gradient boosting framework. It is an enhanced gradient boosting algorithm that is both efficient and adaptable for feature selection and classification [12]. This research proposes a sustainable approach for diagnosing asthma using GANs and XGBoost. The proposed technique may handle the data augmentation and feature selection concerns mentioned above while also enhancing asthma diagnosis.

The remainder of this paper is organized as follows. Section 2 describes the materials and related methods of the GAN and XGBoost. Section 3 explains the proposed approach. Section 4 describes the results and includes the discussion, where thorough comparisons are made between the proposed method and other existing approaches. Finally, Section 5 presents the conclusions.

## 2. Materials and Methods

**Figure 1:**
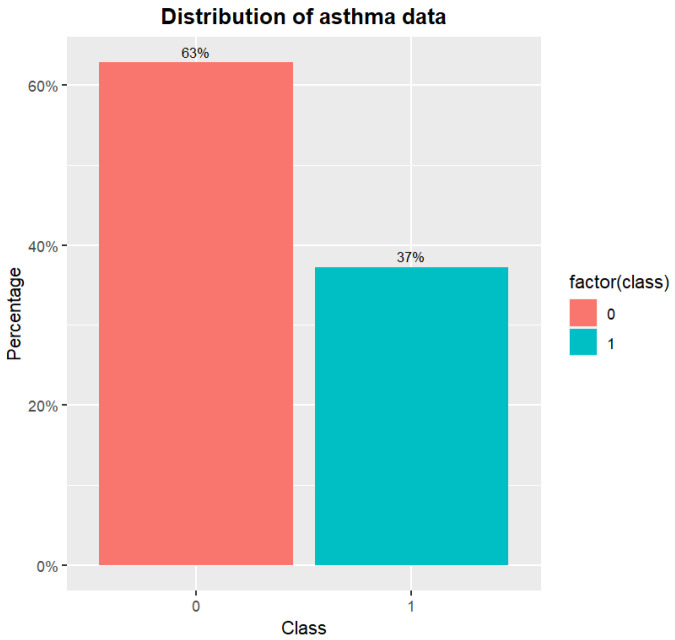
The distribution of asthma data.

**Figure 2:**
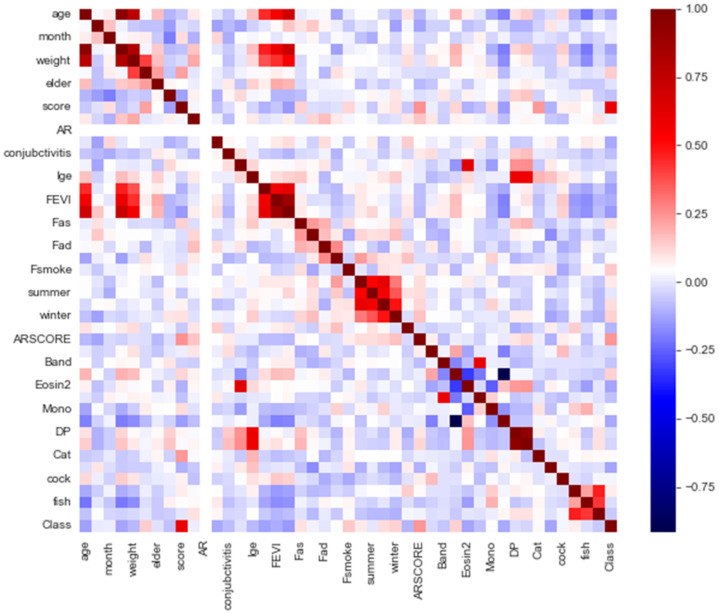
The correlation of features in asthma data.

**Figure 3:**
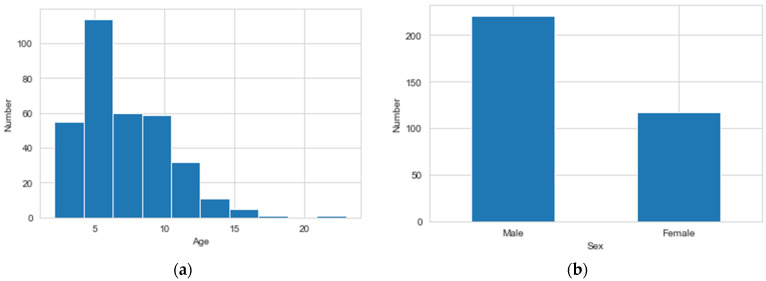
The histogram of the features of Age and Sex. (**a**) The features of Age; (**b**) The features of Sex.

The content was clinical data of asthma patients collected over two years from Taiwan. In this study, the inclusion criteria for patient data were individuals diagnosed with asthma based on established clinical criteria. Patients with incomplete or inconsistent medical records were excluded from the study. Ethical considerations were paramount in this research. All data used in this study were anonymized to protect patient privacy. This study includes 338 asthma samples with 45 features. Table 1 lists the traits of asthma data. The target feature class = 0 represents a patient who does not have asthma (e.g., asthma-negative cases), and class = 1 indicates that the patient is asthmatic (e.g., asthma-positive cases). Figure 1 shows the distribution of original asthma data. It exemplifies the phenomena of skewed data for asthma. The samples of class 0 outnumber those of class 1. Figure 2 depicts the correlation between features in asthma data. Figure 2 shows that the feature Score has the highest association with the target feature class. The wheezing symptom scores are provided by clinicians as the foundation for clinical or routine telehealth care. Figure 3 depicts a histogram of Age and Sex features. The minimum value for the Age feature is 2.12, while the maximum value is 23, and it also shows that there are more males than females.

Because this paper is based on a GAN and XGBoost, in this section, the related GAN and XGBoost are briefly described.

### 2.1. A Brief Description of GANs

**Figure 4:**
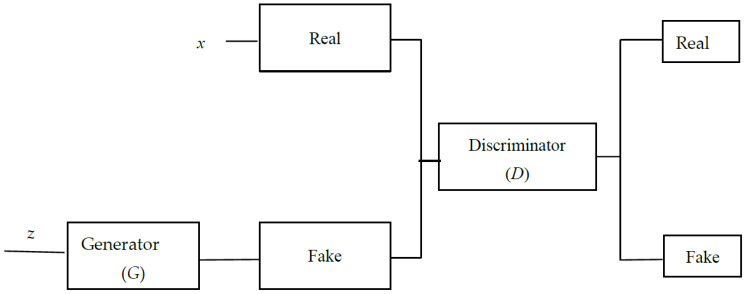
The architecture of GANs.

Goodfellow et al. proposed the GAN model, which is a deep learning model made up of a generator and a discriminator network [13]. According to the discriminator network *D*(*x*), the generator network *G*(*z*) generates fake samples that look like real samples. *z* is a random noise, while *x* is the real sample. The produced sample *G*(*z*) and the real sample *x* are fed into the discriminator network *D*, and the probability of the sample is derived from the real data. The GAN model is optimized using a minimal maximization problem. The GAN model’s cost function is described below:

(1) $$\underset{G}{\min}\underset{D}{\max}V\left(D,G\right)=E_{x\sim p\left(x\right)}\left[\log(D\left(x\right)\right]+E_{z\sim p\left(z\right)}\left[\log\left(1-D\left(G\left(z\right)\right)\right)\right]$$

where *p*(*x*) is the real sample distribution, *p*(*z*) is the noise distribution, and *E*(∙) denotes the calculated expectation. The GAN model in Equation (1) comprises the discriminator optimization process in Equation (2) as well as the generator optimization process in Equation (3). Figure 4 depicts the architecture of GANs.

(2) $$\underset{D}{\max}V\left(D,G\right)=E_{x\sim p\left(x\right)}\left[\log(D\left(x\right)\right]+E_{z\sim p\left(z\right)}\left[\log\left(1-D\left(G\left(z\right)\right)\right)\right]\;$$

(3) $$\underset{G}{\min}V\left(D,G\right)=E_{z\sim p\left(z\right)}\left[\log\left(1-D\left(G\left(z\right)\right)\right)\right]$$

**Figure 5:**
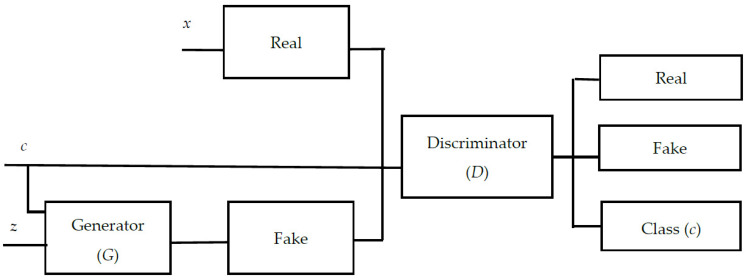
The architecture of ACGANs.

The function of the original GAN network is relatively simple: input noise data, and output fake pictures. With conditional GANs (CGANs), it was then found that precise control of generating samples can be achieved by adding conditional information to the GAN generator. The auxiliary classifier GAN (ACGAN) introduced is a further extension of the CGAN, using the auxiliary classifier to make the GAN obtain a classification function [14]. The architecture of the ACGAN is shown in Figure 5. In Figure 5, each generated sample is assigned a class label *c* in addition to the noise *z*. This class label enables the model to generate data based on the label given. The ACGAN uses the softmax function to make the GAN obtain a classification function. However, the softmax function may be unsuitable for real-world classification. The softmax function used in the ACGAN is indeed a commonly used activation function for multi-class classification problems. However, it may have some disadvantages, particularly in real-world classification scenarios. The softmax function can lead to vanishing gradients. This can slow down or hinder the learning process, making it harder for the model to converge. Softmax normalizes the output scores, ensuring that they sum up to 1. This normalization can sometimes lead to a loss of information, especially when dealing with imbalanced datasets or when certain classes are rare. The output of softmax is a set of probabilities, which implies that the model is confident in its predictions. In real-world scenarios, however, it might be beneficial to have a model that can express uncertainty or assign lower probabilities to less certain predictions [15,16]. This work proposes an enhanced GAN to address the difficulties with ACGANs.

### 2.2. A Brief Description of XGBoost

The XGBoost approach is a popular tree learning algorithm [17,18]. The second-order Taylor expansion of the objective function, which XGBoost uses to ensure model validity, is the system’s core component. It matches the prior forecast’s residuals by repeatedly splitting a new tree. By traversing randomly selected features, XGBoost improves the random sampling ratio, decreases wasted time and overfitting caused by visiting all features, and prevents the tree from becoming too deep by establishing its maximum depth and threshold. The XGBoost model is created in the following manner. To train the model, a first tree is built using the training data, and the difference between expected and actual model values is calculated. After that, until the learning process is complete, a tree is constructed for each iteration to fit the residuals predicted by the model. As a result, a collection of iterative residual trees is created, which are then integrated using multiple tree models. The predicted value ${\hat{y}}_{i}$ is defined as

(4) $${\hat{y}}_{i}=\sum_{k=1}^{K}f_{k}\left(x_{i}\right)$$

where *f_k_*(*x_i_*) represents the expected value of the *k*-th tree, *K* stands for all established trees, and *x_i_* represents the characteristics of the *i*-th sample. The objective function of XGBoost is computed as follows:

(5) $$O_{bj}=\sum_{i=1}^{m}l\left(y_{i},{\hat{y}}_{i}\right)+\sum_{k=1}^{K}\theta \left(f_{k}\right)$$

where *m* represents the total amount of sample data fed into the *k*-th tree. The difference between the true and predicted values of *y_i_* is assessed by the loss function, which is the initial term. The second term is a regularization term, which helps to prevent overfitting and reduces model complexity. Each tree’s complexity is described as follows.

(6) $$\theta \left(f\right)=\beta T+\frac{1}{2}\lambda \;{\left\|W\right\|}^{2}$$

The formula for calculating node segmentation complexity is *β*, where *T* indicates the number of leaf nodes. The L2 regularization coefficient (*λ*) prevents overfitting, whereas *W* indicates the modulus of the leaf node vector.

## 3. The Proposed Algorithm

**Figure 6:**
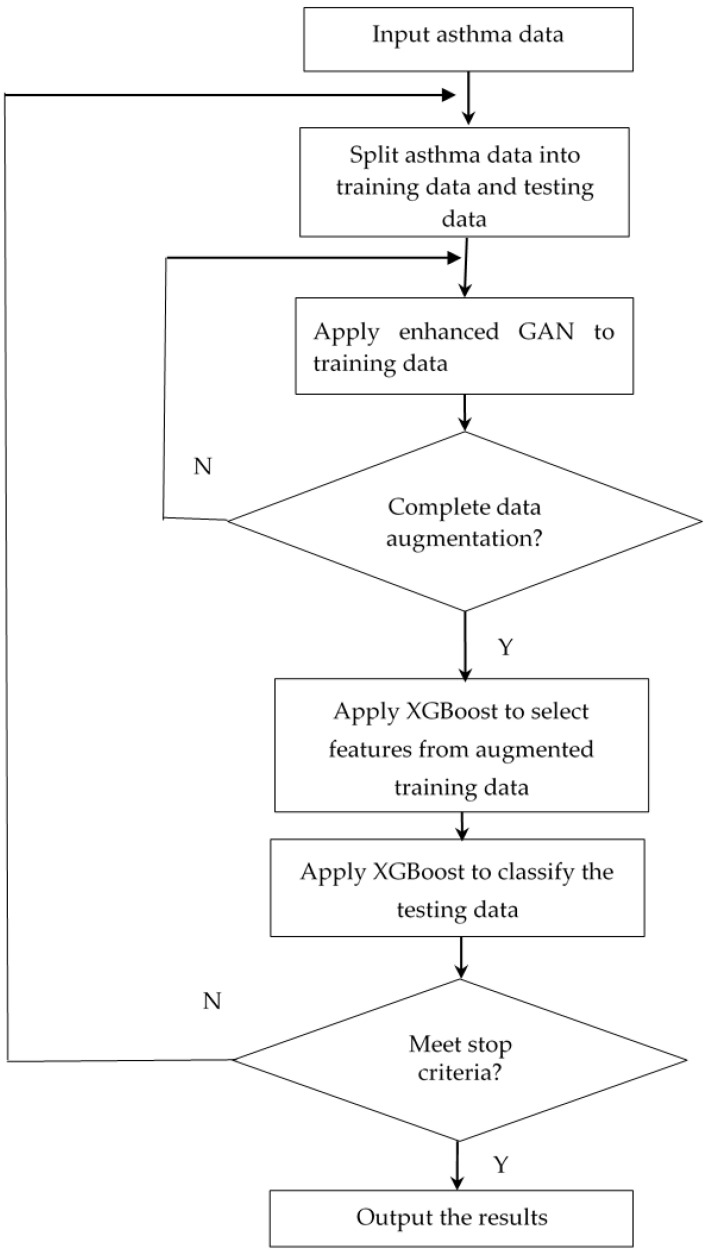
The flowchart of the proposed algorithm.

Asthma datasets often suffer from class imbalance, where one class is underrepresented compared to the other class. This imbalance can lead to biased models that perform poorly in the minority class. To address this issue, we employ enhanced GAN for data augmentation. By training the enhanced GAN on the asthma dataset, we can generate synthetic samples for the minority class, effectively balancing the dataset and improving the performance of the classification model. Feature selection is crucial for building a robust and interpretable asthma diagnosis model. It helps identify the most relevant features that contribute to the prediction of asthma. XGBoost is a powerful machine learning algorithm that is well-suited for feature selection tasks. XGBoost builds a series of decision trees, each focusing on predicting the errors of the previous trees, thereby capturing complex relationships in the data. In this paper, an enhanced GAN- and XGBoost-based intelligent algorithm is proposed to diagnose asthma. The proposed algorithm embeds XGBoost into the GAN as the enhanced GAN to augment the data. Furthermore, it proposes feature selection to increase the performance of asthma classification. Figure 6 depicts the flowchart for the suggested approach. In Figure 6, the asthma data are imported first and then they are split into training data and testing data. The enhanced GAN is used for data augmentation on the training data. When the data augmentation is complete, XGBoost is used to choose features and achieve the highest classification accuracy. The proposed algorithm runs until the stop criteria are met. Finally, the suggested algorithm will output the results.

**Figure 7:**
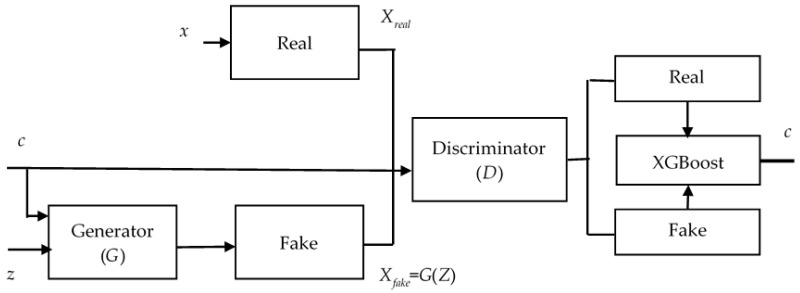
The architecture of enhanced GAN.

The primary network topology of the enhanced GAN remains a generator and discriminator. The generator’s input contains both classification information *c* and random noise *z*. Figure 7 shows that the discriminator’s output includes the corresponding clustering discriminant as well as the probability of whether the data are real or fake. The XGBoost can improve the data classification accuracy while also addressing the issue of insufficient data for analysis. In the loss function of the enhanced GAN, *x* is the raw data, the goal of the discriminator is to maximize *L_x_* + *L_c_* and the goal of the generator is to minimize *L_x_* + *L_c_*.

(7) $$L_{x}=E\left[\log P\left(x=real|X_{real}\right)\right]+E\left[\log P\left(x=fake|X_{fake}\right)\right]$$

(8) $$L_{C}=E\left[\log P\left(c=real|X_{real}\right)\right]+E\left[\log P\left(c=fake|X_{fake}\right)\right]$$

The objective of feature selection is to identify the most effective and essential features required to accurately diagnose asthma. It is well recognized that not every feature in a dataset may hold equal significance. Certain characteristics could give false information that is seen as superfluous or unnecessary. Particularly for the three types of weight, gain, and cover, the feature importance outcomes produced by XGBoost are directly correlated with the model parameter of the importance type. The weight is a measure of how frequently a feature has been applied to models. Because a feature contributes more to the final prediction result when it is utilized in more trees, the weight parameter for a feature indicates its importance. With regard to gain, this refers to the average feature gain towards the expected outcome across all trees. This option represents the feature’s capacity to divide at each node. A feature’s splitting power at each node determines how much it contributes to the ultimate prediction outcome. Cover is the average coverage of a feature in the sample across all trees. This parameter measures the feature’s capacity to cover the model. The significance of a feature in the final prediction result is proportional to its impact on more samples. To choose the most essential features, we employ three parameters introduced in XGBoost. The procedure for feature selection is illustrated below.

Step 1: Perform XGBoost with the specified weight, gain, and cover for feature selection.
Step 2: Select the most essential XGBoost features for each type.
Step 3: Select the same most essential features from each type to represent the feature selection results.
Step 4: XGBoost is used to classify asthma based on the previously selected features.

## 4. Results and Discussions

The *m*-fold cross-validation method is applied in the experiments, with *m* = 5. The dataset is randomly partitioned into five equal-sized subsamples. Of the five subsamples, a single subsample is retained as the validation data for testing the model, and the remaining four subsamples are used as the training data. The cross-validation process is then repeated five times. The proposed algorithm’s convergence serves as the primary stop criterion. It is determined that there is no improvement in the classification accuracy over 30 iterations. In addition, we built protections inside the algorithm to avoid divergent behavior. These protections included restricting the maximum number of iterations to 500.

**Figure 8:**
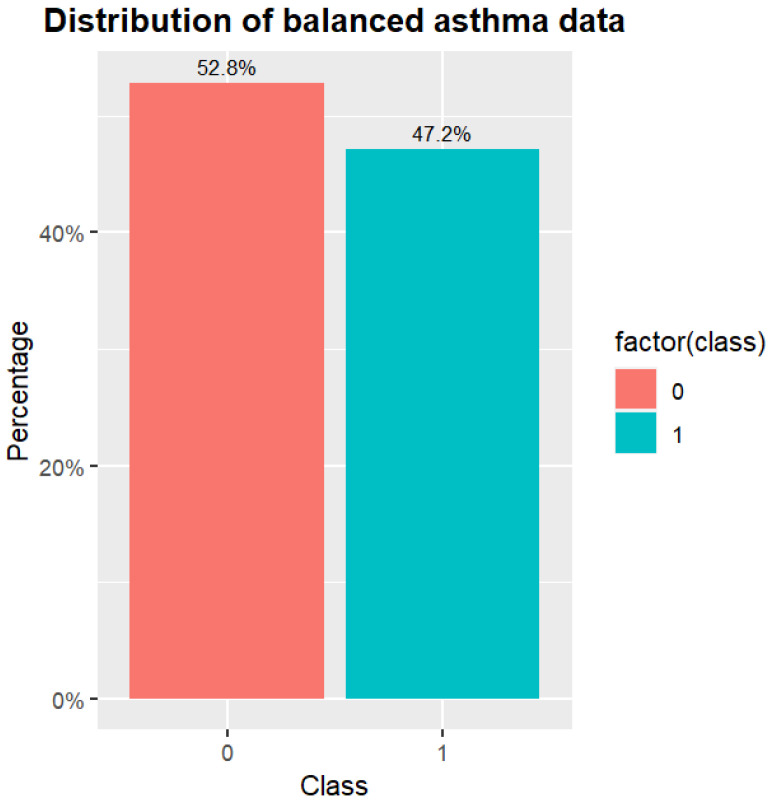
The distribution of balanced asthma data.

After the enhanced GAN, the asthma data are shown in Figure 8. The distribution of the asthma data is more equilibrated than that of the original data. The top five most important features based on feature selection are the features of Score, Eosin2, Fas, Lym, and Mono. The feature of Score is also the most important feature of the correlation. The feature of Eosin2 represents the eosinophilic leukocyte blood draw results. The feature of Fas represents the number of the patient’s family members that suffers from asthma. The feature of Lym represents the lymphatic white blood cell blood draw results. Finally, the feature of Mono represents the mononuclear leukocyte blood draw results.

Several methodologies are compared to validate the suggested algorithm’s performance. The results are presented in Table 2. Based on the simulation findings, the suggested algorithm improves the classification accuracy of classic data mining and machine learning techniques including XGBoost, bacteria foraging optimization with robust fuzzy algorithm (BFO with RFA), support vector machines (SVMs), genetic algorithms (GAs), decision trees (DTs), and backpropagation networks (BPNs). BFO with RFA is an intelligent algorithm based on BFO and RFA, respectively, to analyze asthma [19]. DTs use the partition information entropy minimization algorithm to recursively partition the dataset into smaller subdivisions that are then translated into tree structures [20]. BPNs are among the most widely used artificial neural networks [21]. GAs employ a population of solutions from which increasingly superior solutions can be derived via recombination and selection processes [22]. SVMs are learning algorithms that employ a hypothesis space of linear functions in a high-dimensional feature set [23]. It is noted that XGBoost is without feature selection. The results demonstrate varying classification accuracies among different algorithms, while BPNs, GAs, DTs, and SVMs show relatively lower accuracies at 80.68%, 83.67%, 87.40%, and 89.73%, respectively. Because these above methods are traditional machine learning algorithms, their accuracy is not good enough. XGBoost and BFO with RFA achieve better accuracies, at 90.25% and 91.17%, respectively. The proposed method for asthma diagnosis has a classification accuracy of 94.03%. These results suggest that the proposed algorithm outperforms the other methods in terms of accuracy, indicating its potential superiority in asthma diagnosis.

**Figure 9:**
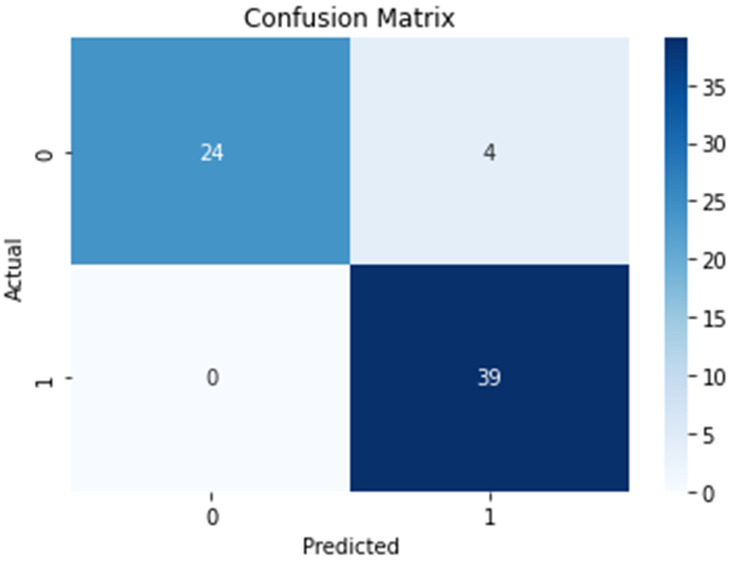
The results of the confusion matrix.

**Figure 10:**
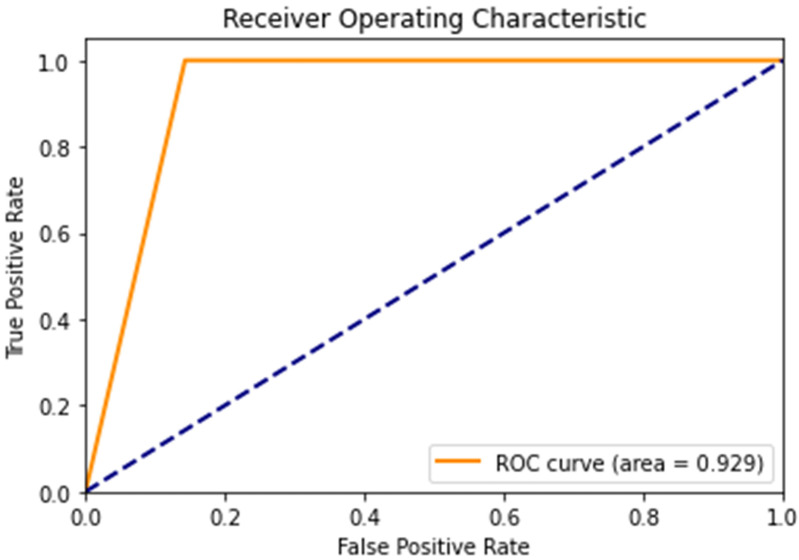
The results of the ROC curve.

For the results, we also use the confusion matrix, a receiver operating characteristic (ROC) curve, and the area under the curve (AUC) metric to evaluate the performance of the proposed algorithm with the testing data of asthma diagnoses. The confusion matrix is shown in Figure 9. It represents a binary classification scenario where 39 patients are correctly predicted as positive, 24 patients are correctly predicted as negative, 4 patients are incorrectly predicted as positive, and 0 samples are incorrectly predicted as negative. From the results, 4 patients had no asthma but were incorrectly diagnosed as asthmatic. In addition, the proposed algorithm correctly diagnosed all the asthmatic patients in the dataset. In other words, there are no false negatives in the predictions. Figure 10 shows the ROC curve. From the ROC curve, the AUC value is 0.929. The AUC value ranges from 0 to 1, where 1 represents a perfect classifier and 0.5 represents a classifier that performs no better than randomly. The AUC value of 0.929 indicates that the proposed algorithm has a strong ability to distinguish between asthmatic and non-asthmatic patients. Overall, the AUC value of 0.929 is considered to be good and indicates that the proposed algorithm is performing well for the given classification task.

## 5. Conclusions

Asthma has long been a national health concern, and it is a common cause of people being unable to work or children missing school. Its morbidity and mortality rates rise year after year in most parts of the world. As can be seen from the collected data, asthma symptoms could begin during early childhood. Most asthmatic children continue to have an atopic constitution with sensitivity to specific allergens being a significant risk factor. The proposed method classifies asthma data with a classification accuracy of 94.03% and an AUC value of 0.929. A comparison of the results to those from other methodologies reveals that the proposed method increases the overall classification accuracy outcomes.

This research describes enhanced GAN- and XGBoost-based approaches to diagnose asthma. The proposed method enables the use of an enhanced GAN to augment data. Then, we use XGBoost to rank the features based on their importance scores. The top-ranked features are selected for training in the final classification model, improving its efficiency and interpretability. Once the data augmentation and feature selection steps are completed, we use a boosting algorithm for the final classification task. Boosting is an ensemble learning technique that combines multiple weak learners to create a strong learner. In our approach, we utilize the XGBoost algorithm again for classification. XGBoost’s ability to handle complicated datasets and its efficiency in handling large datasets make it an ideal choice for asthma diagnosis. By leveraging the power of XGBoost, we aim to build a highly accurate and efficient asthma diagnosis model that can contribute to sustainable healthcare practices.

The feature selection can help find the most important features. The five most significant features are Score, Eosin2, Fas, Lym, and Mono. The top-ranked feature is Score, which also has the highest correlation with the target variable. For the clinical relevance of the top five features, the feature of Score represents the wheezing symptom scores provided by clinicians as the foundation for clinical or routine telehealth care. Wheezing symptom scores are ratings or assessments provided by clinicians to quantify the severity or frequency of wheezing in patients with asthma. Wheezing is a common symptom of asthma characterized by a high-pitched whistling sound when breathing. Clinicians use these scores as a fundamental tool in assessing asthma control and guiding treatment decisions. The feature of Eosin2 represents the eosinophilic leukocyte blood draw results, which are a type of white blood cell involved in allergic reactions and asthma. Elevated eosinophil levels in the blood are often associated with allergic asthma and can indicate ongoing inflammation in the airways. The feature of Fas represents the number of the patient’s family members who suffer from asthma. This feature could be relevant because asthma has a genetic component, and individuals with a family history of asthma are more likely to develop the condition themselves. The feature of Lym represents the lymphatic white blood cell blood draw results. Lymphocytes are a type of white blood cell that plays a crucial role in the immune response. Changes in lymphocyte levels could be indicative of an immune response related to asthma or other conditions. The feature of Mono represents the mononuclear leukocyte blood draw results, which include monocytes and lymphocytes. Monocytes are a type of white blood cell that can differentiate into macrophages, which play a role in the inflammatory response associated with asthma. In summary, these features include indicators of allergic response (Eosin2), genetic predisposition (Fas), immune response (Lym, Mono), and wheezing symptom scores in predicting asthma.

Our algorithm is designed to support healthcare professionals in diagnosing asthma by analyzing patient data and identifying patterns indicative of the condition. While the algorithm demonstrates a 94.03% accuracy in diagnosing asthma, its intent is not to replace clinical judgment but to augment it. By providing healthcare professionals with additional information and insights, the algorithm can help improve the accuracy and efficiency of asthma diagnosis. In terms of patient care, the algorithm has the potential to significantly impact clinical practice. By improving the accuracy of asthma diagnosis, the algorithm can help ensure that patients receive timely and appropriate care. Additionally, early therapies and better patient outcomes may result from the algorithm’s capacity to detect asthma cases that conventional diagnostic techniques might have overlooked. From a clinical workflow perspective, the algorithm seamlessly integrates into existing practices. Healthcare professionals can easily incorporate the algorithm’s results into their diagnostic and treatment decision-making processes. This integration not only streamlines the diagnostic process but also enhances the overall quality of care provided to asthma patients. Our algorithm could reduce the need for unneeded diagnostic tests and treatments, which improves its cost-effectiveness and environmental sustainability. By enhancing the efficiency and effectiveness of asthma diagnosis, our algorithm helps to achieve the larger goals of healthcare sustainability and patient care.

## 6. Future Research Directions

Asthma manifestations in different populations may not be adequately represented in the data, as they were gathered from a Taiwanese healthcare facility. Any clinical or demographic traits of the study population that might be different from those of other groups affect the model’s generalizability. To evaluate the model’s generalizability, validation studies utilizing datasets from other populations or healthcare settings must be carried out. Additional research is encouraged to validate the model’s predictions clinically. This research should include prospective studies or real-world implementation studies to evaluate the model’s effects on asthma diagnosis and treatment.

## Figures and Tables

**Table 1:** The traits of asthma data.

Number	Feature Name	Type	Note
1	The feature of Age	Numerical value	Age of the patient
2	The feature of Sex	Nominal value	The gender of the patient
3	The feature of Month	Numerical value	Patient’s month of birth
4	The feature of Height	Numerical value	Patient’s height
5	The feature of Weight	Numerical value	Patient’s weight
6	The feature of Brothers	Numerical value	Sick with several siblings
7	The feature of the Elder	Numerical value	Elderly people in the patient’s household
8	The feature of Delivery	Nominal value	Mode of birth
9	The feature of Score	Numerical value	Wheezing symptom scores
10	The feature of BW	Numerical value	Weight at birth
11	The feature of AR	Nominal value	Whether the patient has rhinitis or not
12	The feature of AD	Nominal value	Whether the patient has dermatitis or not
13	The feature of Conjunctivitis	Nominal value	Whether the patient has conjunctivitis or not
14	The feature of Eosin	Numerical value	Eosinophilic bulbs
15	The feature of Ige	Numerical value	Immunoglobulin
16	The feature of PEFR%	Numerical value	Lung function
17	The feature of FEVI	Numerical value	Atmospheric tubes
18	The feature of FEF25-75%	Numerical value	Small trachea
19	The feature of Fas	Numerical value	The number of patient’s family suffers from asthma
20	The feature of Far	Numerical value	Whether the family has rhinitis or not
21	The feature of Fad	Nominal value	Whether the family has dermatitis or not
22	The feature of Furticaria	Nominal value	Familial urticarial
23	The feature of Fsmoke	Nominal value	Someone in the family smokes
24	The feature of Spring	Nominal value	Onset season:spring
25	The feature of Summer	Nominal value	Onset season: Summer
26	The feature of Fall	Nominal value	Onset season: autumn
27	The feature of Winter	Nominal value	Onset season: winter
28	The feature of Easiscore	Numerical value	Total score of dermatitis erosion
29	The feature of Arscore	Numerical value	Total rhinitis score
30	The feature of WBC	Numerical value	Total number of white blood cells result from blood draw
31	The feature of Band	Numerical value	Immature white blood cell results from blood draw
32	The feature of Neut	Numerical value	Neutral white blood cell results from blood draw
33	The feature of Eosin2	Numerical value	Eosinophilic leukocyte results from blood draw
34	The feature of Baso	Numerical value	Basophilic leukocyte results from blood draw
35	The feature of Mono	Numerical value	Mononuclear leukocyte results from blood draw
36	The feature of Lym	Numerical value	Lymphatic white blood cell results from blood draw
37	The feature of DP	Numerical value	The results of European dust chamber
38	The feature of DF	Numerical value	The results of American dust chamber
39	The feature of Cat	Numerical value	Whether the patient is allergic to eggs or not
40	The feature of Dog	Numerical value	Whether the patient is allergic to dogs or not
41	The feature of Cock	Numerical value	Whether the patient is allergic to cockroaches or not
42	The feature of the Egg	Numerical value	Whether the patient is allergic to eggs or not
43	The feature of Fish	Numerical value	Whether the patient is allergic to fish or not
44	The feature of Milk	Numerical value	Whether the patient is allergic to milk or not
45	The feature of Class	Nominal value	The target variable

**Table 2:** The classification accuracy for asthma data.

Method	Classification Accuracy
BPN	80.68%
GA	83.67%
DT	87.40%
SVM	89.73%
XGBoost	90.25%
BFO and RFA [19]	91.17%
The proposed algorithm	94.03%

## Data Availability

The utilized data will be supplied upon request with the approval of the healthcare facility.
